# An Account of a Horn, Six and Three Quarter Inches in Length, Developed upon the Head of a Woman

**Published:** 1853-04

**Authors:** Austin L. Sands

**Affiliations:** Cold Spring, N. Y.


### ARTICLE XX.

*An Account of a Horn, six and three quarter inches in length, developed upon the Head of a Woman.* By Austin L. Sands, M. D., of Cold Spring, N. Y.

In the spring of 1851, I was called to see Mrs. W., aged fifty years. She had always enjoyed good health, and was naturally of a full habit. She informed me that she had a "horn" on the back of her head, which had caused her much trouble and uneasiness for a long time, owing to which circumstance she desired its removal. On removing the covering which she kept continually over it, a fine specimen of ichthyosis cornea, of sixteen years growth, was at once brought into view. On inspection, it was found to arise from, or rather immediately over, the occipital protuberance, and to extend downwards and backwards about four inches, and then curling upon itself, terminating in a rough sulcated extremity. On handling it, it was found to be very hard, solid, and, when struck with the handle of a scalpel, gave a sharp clear sound; its attachments to the occiput did not appear to be very firm, as they allowed slight motion. Seeing no difficulty in the way of its immediate removal, an eliptical incision was made on each side of it through the integuments down to the periosteum, and then slipping the scalpel between the base of the horn and the bone, it was easily removed. There was little hemorrhage, and the wound healed kindly by first intention.

On examination after removal, the horn was found to measure *six and three quarter* inches in length, and *three* inches in circumference at the base.

One very interesting point in the process of this case may be found in the history which she gave of the treatment which had been pursued in order to obtain relief. When first perceived, it felt like a shot underneath the integuments; after some time it made its appearance on the surface of the skin, and several times she picked it off, but after a while it became so firm that she was obliged to allow it to remain. She took no notice of it for some time, but, becoming so large as to interfere with the proper adjustment of her cap, and obliging her to raise her head from the pillow at night whenever she wished to turn over in bed, while it also incommoded her from reposing upon her back, she was necessitated to apply to her family physician (a homeopath) for relief, who promised her that in the space of a short time he would be enabled to remove the difficulty by the use of sundry small white pills which he proceeded to furnish her with; while at the same time he assured her that he had treated successfully, to a cure, several similar cases within the past year in his own practice. For five years she continued to use the homeopath's pills, but still the *horn* remained—still the horn continued to grow. In view of this condition of affairs her faith began to falter, but for some time it was supported with the assurance of her physician that the only reason that the cure had not been effected, was, "that he had not as yet got hold of the right pills." Reassured by this assertion, which was repeated from time to time, she kept on until the spring of the present year, when she consulted me, and finally submitted to its removal by the knife.—*N. Y. Journal of Medicine.*

